# Vitamin C Intake and Risk of Prostate Cancer: The Montreal PROtEuS Study

**DOI:** 10.3389/fphys.2018.01218

**Published:** 2018-09-04

**Authors:** Marie-Elise Parent, Hugues Richard, Marie-Claude Rousseau, Karine Trudeau

**Affiliations:** ^1^INRS-Institut Armand-Frappier, Université du Québec, Laval, QC, Canada; ^2^School of Public Health, Université de Montréal, Montréal, QC, Canada; ^3^Centre de Recherche du Centre Hospitalier de l’Université de Montréal, Montréal, QC, Canada

**Keywords:** vitamin C, prostate cancer, diet, supplements, case-control, population-based, etiology, prevention

## Abstract

**Background:** Vitamin C is a reducing agent and free radical scavenger, acting as antioxidant in plasma membranes and within cells. Based on these properties, a role for vitamin C in cancer incidence has been suspected. There are as yet few large population-based studies focusing on prostate cancer, with the preponderant evidence leaning toward the absence of an association. Nevertheless, many previous studies overlooked prostate cancer aggressiveness, as well as screening and detection issues, which could bias potential associations.

**Methods:** The Prostate Cancer and Environment Study (PROtEuS) is a population-based case-control study conducted in Montreal, Canada. In-person interviews, conducted with 1,916 histologically confirmed prostate cancer cases and 1,985 population controls, elicited information on a wide range of socio-demographic, lifestyle, and medical factors, including PSA screening. Usual frequency of consumption of 63 food items two years prior to diagnosis/interview was collected, along with use of dietary supplements. Odds ratios (OR) and 95% confidence intervals (CI) between vitamin C intake and prostate cancer were estimated using logistic or polytomous regression, adjusting for potential confounders.

**Results:** We observed no association between dietary intakes of vitamin C (OR for upper vs. lower tertile: 0.95, 95%CI 0.77, 1.18), estimated using the residual method to account for energy intake, or between regular use of vitamin C supplements and/or multivitamins (OR 0.90, 95%CI 0.76–1.05), and overall prostate cancer. Analyses considering disease aggressiveness, or restricted to subjects recently screened with PSA, thereby limiting the potential for undiagnosed cancers in non-cases, generated results consistent with those from the main analyses.

**Conclusion:** Our findings document the absence of an association between recent dietary vitamin C intake, or supplementation, and prostate cancer incidence overall or prostate cancer grade at diagnosis. Based on this, and other available evidence, vitamin C intake does not seem to hold promises with regard to prostate cancer prevention.

## Introduction

Prostate cancer is the most frequently diagnosed solid-tumor cancer among men in the world (Ferlay et al., 2013). The only clearly established factors to date are age, a family history of the disease and ancestry. The identification of modifiable risk factors, which would be amenable to prevention, would represent a major scientific achievement. The suspicion that diet plays a role in prostate cancer development has led to the conduct of several studies on this issue. In its 2014 update on prostate cancer, the World Cancer Research Fund reported on the evidence for several dietary factors (WCRF and AICR, 2014). Based on studies at the time, there was limited evidence, albeit suggestive, that high intakes of dairy products, and low plasma levels of alpha-tocopherol and selenium, are associated with increased risk of prostate cancer. For vitamin C, data were judged to be either of too low quality, too inconsistent, or the number of studies too few for conclusions to be reached. This led to the classification of vitamin C, in relation to prostate cancer, as “Limited-no conclusion." Since then, two studies focusing on dietary supplements have been published. A Swedish case-control study observed no association with use of vitamin C supplements and prostate cancer overall, or by aggressiveness (Russnes et al., 2016). Moreover, in a US randomized trial, vitamin C supplementation had no effect on the risk of overall or lethal prostate cancer (Wang et al., 2014).

A salient characteristic of prostate cancer is that some cancers are aggressive, with a poorer prognosis, while others are not. The reason for this remains elusive. Aggressive cancers are particularly important from a clinical standpoint whereas identification and treatment of unaggressive ones bear consequences that significantly alter the quality of life. There is indeed mounting evidence that these two types do not necessarily share the same risk factors. For example, body fatness appears to be related to advanced prostate cancer more specifically (WCRF and AICR, 2014). Moreover, there is evidence that alcohol intake might be associated with high-grade prostate cancer and not with the less aggressive form of the disease (Demoury et al., 2016). This points out to the need to consider tumor aggressiveness in etiological studies of prostate cancer. To our knowledge, only a handful of studies investigating the potential association between vitamin C and prostate cancer have done so (Andersson et al., 1996; Cohen et al., 2000; Kristal et al., 2010; Roswall et al., 2013; Russnes et al., 2016).

Another major caveat of previous investigations of diet in prostate cancer is that many overlooked important features of the disease. One such issue relates to cancer detection. Many prostate cancers are latent and asymptomatic. Epidemiological studies have rarely considered the possible presence of prostate cancer in subjects considered as non-cases in cohort or case-control studies. A misclassification of outcome could have led to a decreased opportunity to observe associations. Moreover, not taking into account prostate cancer screening practices might have obscured associations. For instance, adherents to a healthy diet and lifestyle may be more prone to seek prostate cancer screening. Conversely, individuals under close medical follow-up for an illness that may be related to diet might be screened more frequently, and thus be more likely to be diagnosed with prostate cancer. In both situations, if screening patterns are not considered, the associations observed will be distorted (Giovannucci, 2007; Garcia-Closas and Berrington de Gonzalez, 2015).

In order to provide new evidence on this issue, we examined the relationship between dietary intake of vitamin C, as well as of intake of vitamin C supplements and multivitamins, and the risk of incident prostate cancer in a large population-based case-control study, specifically addressing the issues of cancer aggressiveness and screening patterns.

## Materials and Methods

### Study Population and Data Collection

#### Study Population

The Prostate Cancer & Environment Study (PROtEuS), described previously (Blanc-Lapierre et al., 2015), is a population-based case-control study conducted in Montreal, Canada, to assess the role of environmental and lifestyle factors in prostate cancer risk. Eligible subjects were men, younger than 76 years of age at the time of diagnosis or selection, residents of the greater Montreal area, registered on Quebec’s permanent electoral list (continually updated) and Canadian citizens.

Cases were all patients newly diagnosed with primary histologically confirmed prostate cancer, actively ascertained through pathology departments across seven French-speaking hospitals in the Montreal area between 2005 and 2009. This covered over 80% of all prostate cancers diagnosed in the region of Montreal during the study period according to registry information. Concurrent to case recruitment, controls were randomly selected from the electoral list of French-speaking men residing in the Montreal area and frequency-matched to cases in 5-year age groups.

Study participants represented 79.4% of eligible cases and 55.5% of eligible controls. For less than 4% of subjects who were not available (e.g., deceased or too ill), the interview was conducted with a proxy respondent, usually the spouse. Reasons for non-participation, among cases and controls, were refusal (94 and 86%), unable to trace (3 and 11%), death (2 and 1%) or too sick to participate (1% of controls) with no proxy available, and language barrier (1 and 1%), respectively.

#### Data Collection

During face-to-face interviews, subjects provided information on socio-demographic and anthropometric characteristics, and medical factors such as history of type II diabetes and family history of cancer. The screening history by prostate-specific antigen (PSA) in the recent 5 years was elicited. Information was also collected about lifestyle factors such as lifetime alcohol consumption, smoking habits, and diet. Tumor grades, defined by the Gleason score, were extracted from prostate biopsy pathology reports.

This study was approved by the Ethics Committees of the following institutions: Institut national de la recherche scientifique, Centre de Recherche du Centre Hospitalier de l’Université de Montréal, Hôpital Maisonneuve-Rosemont, Hôpital Jean-Talon, Hôpital Fleury, and Hôpital Charles-LeMoyne. All subjects provided written informed consent.

### Dietary Intake

Diet was assessed using a food frequency questionnaire (FFQ) adapted from the instrument developed by the Canadian Cancer Registries Epidemiology Research Group (Pan et al., 2004), which was based on two extensively validated questionnaires: the National Cancer Institute’s Block Questionnaire (Block et al., 1986) and the Nurses’ Health Study FFQ (Hu et al., 2016). Minor modifications were made to reflect the specificity of the study population (Pan et al., 2004). The FFQ was pre-tested for face-validity in a subgroup of the target population to ensure that questions were well understood. Subjects were asked about their consumption of food at home, at work, and restaurants, two years prior to diagnosis for cases or interview for controls. Data pertaining to lifetime consumption of beer, wine and spirits were also collected.

The FFQ covered 63 food items. Food intake was recorded in terms of the frequency of use per day, week or month of commonly used portions. Given the seasonal variations in consumption of fruits, participants were asked how many months per year they ate various fruits. Additional questions were asked to help refine the intake assessment. These included the frequency of eating fat when consuming beef, pork and chicken, the degree of doneness when eating beef, the typical portion of meat consumed, the frequency of consumption of charred meat, the type of fat usually used for cooking meat, the types of breakfast cereals and of milk most often consumed. In order to assess general changes in consumption patterns over time, subjects were asked whether they tended to consume less, the same or more of 10 food categories, including fruits and vegetables, as compared to 20 years earlier. The nutrient content of foods was extracted from the 2010 Canadian Nutrient File (Health Canada, 2010).

### Vitamin Supplements

Subjects were asked to report whether, throughout their adult life, they had taken vitamin or mineral supplements at least once a week. A list of 12 pre-specified supplements, including vitamin C and multivitamins, along with an open-ended item for supplements not on the list, was provided. Commonly used multivitamins were presumed to have contained vitamin C. For each item, possible answers were “yes, regularly (defined as at least once a week),” “yes, occasionally,” “no,” or “don’t know.”

### Statistical Analyses

Unconditional logistic regression models were used to estimate odds ratios (OR) and 95% confidence intervals (CI) for the risk of overall prostate cancer associated with dietary intake of vitamin C, as well as with vitamin C/multivitamin supplements. Polytomous regression models were applied to estimate the association between vitamin C intake and prostate cancer according to tumor grade. Gleason grades ≥ 8 or 7[4+3] were considered as indicative of aggressive cancers, whereas lower grades were classified as unaggressive (Wright et al., 2009).

Individual daily intakes of micro and macronutrients, including vitamin C, were calculated by multiplying the weekly frequency of intake of each food by the nutrient content of the specified portion size, then summing the contributions from all foods and dividing by seven. These are subsequently referred to here as absolute intakes. A second approach, the residual method, was also applied to derive the predicted intake of each nutrient based on the median daily energy intake among men in the sample (Willett et al., 1997). The latter method reduces measurement error for specific nutrients and removes extraneous variation, allowing the direct evaluation of variation due to dietary composition rather than absolute nutrient intakes. Vitamin C intake values were then categorized into tertiles based on the frequency distribution among controls, established separately for the absolute and residual methods.

In all, 17 subjects (0.4%) were excluded from the dietary analyses either because they had not filled in the dietary section or because they had answered less than 50% of the dietary questions. It has indeed been observed previously that it is reasonable to exclude subjects for whom 50% or more of items on a FFQ are left unanswered, if missing values are randomly distributed across the questionnaire (Willett, 1998).

Regression models included the following *a priori* potential confounders: age (continuous), ancestry (European, Asian, Sub-Saharan African, other, and don’t know), first-degree family history of prostate cancer (no, yes, and don’t know), education (elementary, high school, college, university, and other), income in Canadian dollars (< $20,000$, $20,000–29,999, $30,000–49,999, $50,000–79,999$, ≥ $80,000, prefers not to respond/don’t know), physical activity level (not very active, moderately active, very active, and don’t know), body mass index (BMI) two years prior to interview (continuous), type II diabetes (no, yes, and don’t know), frequency of PSA screening in the previous 5 years (0, 1–4, ≥ 5 tests, don’t know), cigarette-years (continuous), respondent status (self, proxy), lifetime beer intake in drink-years (4 categories), daily intakes of red meat (3 categories), saturated fats (3 categories), energy and daily dietary intakes (continuous) of beta-carotene, polyunsaturated fatty acids, alpha-tocopherol, folates, selenium, lycopene, and calcium. Continuous variables were entered as such after confirming linearity of the logit, otherwise they were broken down into categories.

Several sensitivity analyses were conducted: a) limiting the list of potential confounders to those factors clearly recognized as risk factors for prostate cancer, i.e., age, ancestry, family history of the disease, and as well as for energy intake; b) excluding controls not screened for prostate cancer in the previous two years to reduce the likelihood of undiagnosed cancers among controls; and c) restricting analyses to self-respondents.

We could not calculate the total vitamin intake from diet and supplements owing to the semi-quantitative nature of the latter. We thus explored effect modification by regular use of vitamin C supplements and/or multivitamins by including cross products in the multivariate models along with dietary vitamin C intake.

The final sample for analysis consisted of 3,897 subjects, i.e., 1,916 prostate cancer cases and 1,985 population controls. Three cases had missing information on the primary or the secondary Gleason grade and these were excluded from analyses based on disease aggressiveness, leaving 430 high-grade and 1483 low-grade cancers for analysis.

Statistical analyses were performed using SAS version 9.3, SAS Institute Inc., Cary, NC, United States.

## Results

Study participants are described in **Table 1**. Controls were slightly older (64.8 years, on average) than cases (63.5 years) owing to the slightly longer period required to secure interviews with controls. The majority of subjects were of European descent. As expected, based on the only confirmed risk factors for prostate cancer, cases tended to be more often of African origin and less often of Asian descent than controls. Also, about twice as many cases as controls had a first-degree family history of the disease. There were small, albeit not statistically significant, differences between cases and controls in terms of education and family income, overall physical activity level, mean cumulative smoking and beer consumption. In agreement with previous evidence (Kasper and Giovannucci, 2006), type II diabetes was less common among cases. Cases had a slightly lower mean BMI than controls and had undergone more PSA tests in the preceding 5 years.

**Table 1:** Selected characteristics of study subjects.

	Cases (*n* = 1,916)	Controls (*n* = 1,985)	
Age in years, mean ± std	63.5 ± 6.8	64.8 ± 6. 9	<0.0001
**Ancestry, *n* (%)**			<0.0001
African	128 (6.7)	86 (4.3)	
Asian	24 (1.3)	69 (3.5)	
European	1,677 (87.5)	1,683 (84.8)	
Other	75 (3.9)	133 (6.7)	
Don’t know	12 (0.6)	14 (0.7)	
**First-degree family history of prostate cancer, *n* (%)**			<0.0001
No	1,402 (73.2)	1,730 (87.2)	
		196 (9.9)	
Yes	446 (23.3)	59 (3.0)	
Don’t know	68 (3.5)		
**Education, *n* (%)**			0.2522
Elementary	445 (23.2)	426 (21.5)	
High School	568 (29.6)	575 (29.0)	
College	313 (16.3)	375 (18.9)	
University	587 (30.6)	607 (30.6)	
Don’t know	3 (0.2)	2 (0.1)	
**Family income in CAN$, *n* (%)**			0.1453
<20,000	222 (11.6)	243 (12.2)	
20,000–29,999	262 (13.7)	251 (12.6)	
30,000–49,999	444 (23.2)	460 (23.2)	
50,000–79,999	422 (22.0)	410 (20.7)	
≥80,000	424 (22.1)	428 (21.6)	
Prefers not to respond/don’t know	142 (7.4)	193 (9.7)	
**Body mass index 2 years ago in kg/m^2^, mean ± std**	26.8 ± 4.0	27.2 ± 4.4	0. 0019
**Physical activity, *n* (%)**			0.1701
Not very active	430 (22.4)	485 (24.4)	
Moderately active	521 (27.2)	557 (28.1)	
Very active	964 (50.4)	943 (47.5)	
**Smoking in cigarettes-years,^a^ mean ± std**	610.4 ± 539.01	649.8 ± 546.78	0.0549
**Beer consumption in drink-years,^b^ mean ± std**	56.2 ± 101.25	54.9 ± 100.51	0.7500
**History of type II diabetes, *n* (%)**			<0.0001
No	1,684 (87.9)	1,604 (80.8)	
Yes	229 (12.0)	393 (19.0)	
Don’t know	3 (0.2)	3 (0.2)	
**Number of PSA tests in previous 5 years^c^, *n* (%)**			<0.0001
≥5	982 (51.3)	889 (44.7)	
1–4	797 (41.6)	657 (33.1)	
0	6 (0.3)	254 (12.8)	
Don’t know	130 (6.8)	185 (9.3)	
**Respondent status, *n* (%)**			0.0242
Self	1867 (97.4)	1909 (96.2)	
**Daily dietary intakes, mean ± std**			
Vitamin C, mg (absolute amount)	150.8 ± 80.1	145.7 ± 74.8	0.0400
Vitamin C, mg (based on residual method)	145.3 ± 75.7	142.9 ± 70.0	0.3041
Vitamin E, mg	6.7 ± 2.4	6.6 ± 2.5	0.3317
Calcium, mg	1026.4 ± 422.7	1000.3 ± 405.1	0.0493
Selenium, μg	143.9 ± 47.4	141.2 ± 48.1	0.0791
Lycopene, μg	8770.0 ± 7044.2	7826.9 ± 6274.5	<0.0001
Beta-carotene, μg	4420.2 ± 2612.0	4334.7 ± 2820.6	0.3265
Folates, μg	472.1 ± 156.8	458.5 ± 157.1	0.0068
Saturated fatty acids, g	27.0 ± 11.6	26.0 ± 11.6	0.0053
Polyunsaturated fatty acids, g	14.1 ± 5.5	13.9 ± 5.5	0.2007
Red meat, g	132.7 ± 90.4	128.1 ± 93.0	0.1168
Energy, kcal	2243.9 ± 708.6	2172.5 ± 690.2	0.0015
**Regular^d^ use of vitamin C supplement, *n* (%)**	196 (10.2)	181 (9.1)	0.4273
**Regular^d^ use of multivitamin supplement, *n* (%)**	358 (18.7)	392 (19.7)	0.6904

*^a^Among ever smokers. ^b^Among subjects who reported ever consuming alcohol once a month for one year or more ^c^PSA: prostate-specific antigen. ^d^Defined as at least once a week.*

The different foods contributing to vitamin C intake were as follows, in descending order for a typical portion: cantaloupe, orange, red pepper, strawberry, vegetable juice, clementine, tomato, orange juice, broccoli, apple juice, tomato juice, honeydew melon, cabbage, kiwi, banana, grapefruit, tomato ketchup, tomato soup, sweet potato, spinach, coleslaw, apple, peach, mango, pizza (from tomato sauce and vegetable toppings), and vegetable soup.

**Table 1** also presents the mean daily dietary intakes of selected nutrients among cases and controls. Mean absolute dietary vitamin C intakes were higher among cases (151 mg) than controls (146 mg). However, this difference was no longer significant when vitamin C intake was calculated applying the residual method. The mean daily dietary intakes of other nutrients tended to be generally slightly higher among cases than controls, although only for calcium, lycopene, folates, saturated fatty acids, and energy did differences achieve statistical significance. Ten percent of cases and nine percent of controls reported using vitamin C supplements regularly. Corresponding values for multivitamin supplements were 19 and 20%, respectively. Case-control differences in supplements use were not statistically significant.

The ORs for prostate cancer across tertiles of dietary vitamin C intake based on absolute amounts and when applying the residual method are shown in **Table 2**. Irrespective of the approach used, there was no evidence of an association between intake levels and prostate cancer risk. This held true considering all cases or by cancer aggressiveness. No dose-response patterns emerged nor was there evidence of a U-shape relationship.

**Table 2:** Adjusted^a^ OR for the association between dietary vitamin C and risk of prostate cancer, for all cancers and by disease aggressiveness.

Tertiles of intake, mg/day^b^	Controls	All cases			Non-aggressive cases^c^			Aggressive cases^c^		
	*n*	*N*	OR	95%CI	*n*	OR	95% CI	*n*	OR	95%CI
**Model 1 – Dietary intakes expressed as absolute amounts**										
T1: ≤107.7	656	613	(ref.)		471	(ref.)		141	(ref.)	
T2: >107.7–167.6	655	611	0.93	0.78, 1.11	471	0.93	0.77, 1.13	139	0.95	0.71, 1.26
T3: >167.6	674	692	0.95	0.77, 1.18	541	0.98	0.79, 1.23	150	0.89	0.64, 1.24
**Model 2 – Dietary intakes calculated using the residual method**										
T1: ≤109.8	655	638	(ref.)		491	(ref.)		146	(ref.)	
T2: >109.8–164.9	656	609	0.96	0.80, 1.14	465	0.94	0.78, 1.14	143	1.00	0.76, 1.33
T3: >164.9	674	669	0.97	0.79, 1.19	527	0.99	0.80, 1.23	141	0.91	0.66, 1.27

*CI, confidence interval; n, number of subjects; OR, odds ratio; (ref.), reference category. ^a^Adjusted for age (continuous), ancestry (Caucasian, Asian, Sub-Saharan African, other, and don’t know), first-degree family history of prostate cancer (no, yes, and don’t know), education (elementary, high school, college, university, and other), income (5 categories), physical activity level (sedentary, moderately active, and very active), body mass index in kg/m^2^ (continuous), type II diabetes (no, yes, and don’t know), respondent status (self, proxy), number of PSA tests in the previous 5 years (0, 1–4, ≥ 5, and don’t know), cigarette-years (4 categories), beer-years (4 categories), red meat consumption in grams/day (3 categories), saturated fat in grams/day (continuous), energy intake in Kcal/day (continuous), selenium (3 categories), and dietary intakes (continuous) of calcium, lycopene, folates, alpha-tocopherol and beta-carotene. ^b^Tertiles based on the frequency distribution of energy-adjusted vitamin C intake among controls. ^c^Gleason grades ≥ 8 or 7[4+3] were considered as indicative of aggressive cancers, whereas lower grades were classified as non-aggressive cancers. Excludes 3 subjects for whom the primary or the secondary Gleason grade is missing.*

Regular use of vitamin C supplements and/or multivitamins was not associated with prostate cancer risk (OR 0.90, 95%CI 0.76–1.05). Moreover, analyses including a variable for regular use of vitamin C and/or multivitamin supplement and a cross-product term between this variable and dietary vitamin C intake based on the residual method indicated no significant interaction (*p*-value for interaction term = 0.06).

To evaluate the impact of including several lifestyle and dietary factors on risk estimates, we ran additional models including only age, ancestry, family history of the disease, and energy intake. This had only a marginal effect on risk estimates (data not shown). Excluding controls not screened for prostate cancer in the previous two years, and restricting analyses to self-respondents also generated results similar to the main findings (data not shown).

## Discussion

Findings from our study, consistent across different sub-analyses, support the notion that recent vitamin C intake from dietary sources is not associated with incident prostate cancer, irrespective of its aggressiveness. There was no evidence of dose-response or U-shape relationships, and results were unchanged when considering vitamin C or multivitamin supplements.

There has long been an interest in identifying possible lifestyle or environmental factors that could be modified in order to prevent prostate cancer. Evidence from migrant studies and the geographic distribution of the disease indeed suggest that its development is not strictly under genetic influences and that environmental and lifestyle factors likely play an important role (Haenszel and Kurihara, 1968; Haas and Sakr, 1997; Hsing and Chokkalingam, 2006). Defining the actual factors actually involved has, however, proven to be challenging. Several studies investigating the role of dietary factors in prostate cancer risk have been conducted. In a recent review (Lin et al., 2017), low carbohydrate intake, soy protein, omega-3 fatty acids, green tea, tomato products were judged of potential interest for reducing prostate cancer risk and progression. By contrast, there were indications that elevated intakes of animal fat and higher β-carotene status might increase risk. The authors further raised the possibility of a U-shape relationship between intakes of folates, vitamin D, calcium, vitamin C, and prostate cancer risk, whereas both deficiency and high intakes would be associated with increased risk of the disease (Morris and Tangney, 2011).

The mechanisms through which vitamin C could be involved in prostate cancer prevention have been reviewed (Willis and Wians, 2003), mainly involving the protection of cytosolic and cell membranes from oxidative damage. Vitamin C would act primarily in the cytosol of cells by scavenging free radicals generated during cellular metabolism, and indirectly, by protecting cells membranes from free radical-induced damage (Scarpa et al., 1984). *In vitro* studies using androgen-dependent and androgen-independent cell lines have demonstrated that vitamin C causes a dose- and time-dependent decrease in cell number, viability and DNA synthesis (Maramag et al., 1997). Also, the combination of vitamin K_3_ and vitamin C appears to exert a synergistic antitumor effect on certain androgen-dependent cell lines (Jamison et al., 1996; Jamison et al., 2001).

Based on the observation of a dose-dependent relationship in an early and smaller study of vitamin C supplementation (Kristal et al., 1999) it has been proposed that the lack of association in many epidemiological studies of vitamin C intake might be explained by the fact that study subjects may not have had sufficient vitamin C levels to observe such an association (Willis and Wians, 2003). However, subsequent studies of vitamin C supplementation (500 mg daily in the Physician’ Health Study II trial) at doses often higher than those typically encountered in the diet (mean intake of about 150 mg in our study) failed to demonstrate an association between vitamin C and prostate cancer. Dietary intakes in our population were comparable to those (median of 93 mg) observed in a Danish cohort (Roswall et al., 2013).

A meta-analysis including studies published between 1992 and 2013 reported an inverse relationship between dietary vitamin C and risk of prostate cancer (Bai et al., 2015). Both cohort (RR 0.92, 95%CI 0.86–0.99, 6 studies) and case-control (RR 0.80, 95%CI 0.71–0.89, 12 studies) investigations were consistent with reduced risks among men classified in the highest exposure categories. There were, however, several issues with this analysis. For instance, it included two studies of benign prostate hyperplasia and one study focusing on vitamin supplements while these studies were explicitly ineligible based on exposure or outcome. Nevertheless, what stands out from the list of studies included is that of the 15 studies of dietary intake and prostate cancer, nine were based on less than 500 cases, often much less. Moreover, prostate cancer screening was taken into account in only two of these studies and only two studies examined advanced or aggressive prostate cancer.

Investigations subsequent to those included in the meta-analysis include a post-trial follow-up of a randomized trial in the Physicians’ Health Study II (Wang et al., 2014), suggesting no immediate or long-term effects of vitamin C supplementation on the risk of incident or lethal prostate cancer. There was also no evidence that use of vitamin C or multivitamin supplements were associated with prostate cancer (all cancers, deaths, grade and advancement) in a Swedish case-control study including 1499 cases and 1112 controls (Russnes et al., 2016).

Two other meta-analyses have been conducted, this time focusing on vitamin C supplementation. One included two randomized controlled trials (RCT) (Jiang et al., 2010) while the other included one RTC, one cohort and one case-control study (Stratton and Godwin, 2011). Findings were consistent with the absence of an association with prostate cancer risk.

Vitamin C from supplements and diet might differ in terms of absorption and biological activity. However, several studies document little difference in steady-state plasma and/or urine bioavailability of synthetic vitamin C and that found in different fruits, fruit juices and vegetables (Pelletier and Keith, 1974; Mangels et al., 1993; Carr et al., 2013). It may be that users of dietary supplements tend to have more health issues (Bender et al., 1992) or conversely, harbor more health-seeking behaviors (Kirk et al., 1999). We had information on several such factors, thought to relate to prostate cancer risk, including type II diabetes, obesity, physical activity, dietary intake of several nutrients, and prostate cancer screening. Adjusting for these in our models had a minimal influence on risk estimates.

As in any study assessing dietary intakes, measurement error inevitably occurred in the study. Our FFQ was adapted from widely used validated questionnaires (Block et al., 1986; Willett, 1998), which served to develop other FFQs validated and used in various Canadian and Québec populations (Pan et al., 2004; Boucher et al., 2006). As exposure misclassification resulting from the use of the FFQ was likely non-differential with respect to case-control status, this may have resulted in a spurious attenuation of associations. Reporting bias is always of concern in retrospective studies. While the diet questionnaire focused on the period two years prior to diagnosis, reports among cases might have been influenced by their illness when reporting their dietary habits. This could have been in the direction of an inflation of recent intakes among cases, who had increased their fruit and vegetable consumption, or of a reduced intake as the result of illness, treatment or psychological issues. Nevertheless, reporting bias is also possible among controls, who may have been dealing with health conditions other than prostate cancer. We compared the frequency of use of fruit and vegetables in our control series to that of controls in a Canadian-wide population-based study (Villeneuve et al., 1999) and found nearly identical mean and median intakes in both populations.

The food-frequency questionnaire focused on recent diet (two years before diagnosis or interview). This time point may not reflect the relevant etiological period with respect to prostate cancer. Recent intakes would be particularly relevant if vitamin C acted to prevent cancer at the promotion stage of the disease whereas remote intakes would likely be more relevant to cancer initiation. We did not have information on past intake of vitamin C-contributing foods, i.e., fruit and vegetables. However, we had a crude, semi-quantitative assessment as to whether subjects had changed their frequency of use of fruit and vegetables over the last 20 years. Regarding the frequency of fruit use, 52% of subjects indicated using similar levels 20 years earlier, as compared to recent use, while 34% used less and 14% used more in the past. The corresponding figures for vegetables were 50, 28, and 12%, respectively. The proportion of subjects who could not recall whether they had changed their consumption patterns was less than one percent for both food groups. These observations suggest that our intake assessment cannot be thought to reflect accurately vitamin C intake in the remote past, as about half of our subjects indicated having changed their intake patterns over the previous 20 years. Nevertheless, given the timing of our dietary assessment, our finding suggests the absence of a role for vitamin C in prostate cancer at the promotion stage.

The use of vitamin supplements is typically hard to recall and estimate, as these can be used on a sporadic basis and over short periods. Moreover, the contents in vitamin C varies according to the product used. Based on this, our assessment was conducted in a crude way, eliciting prior, regular use of vitamin C and or multivitamin supplements. We can reasonably presume, based on the formulas readily available on the market in the study area, that the multivitamins contained vitamin C, but at lower levels (typically around 100 mg) than in supplements containing exclusively vitamin C (typically 500 mg). Our study compares well to a Swedish population-based study (Russnes et al., 2016), where the proportions of vitamin C (12–15%) and multivitamin (16–22%) supplements users were similar to ours. Both population-based studies found no association between supplements use and prostate cancer risk.

Participations rates were relatively high (79%) in cases and more modest (56%) in controls, raising the possibility of selection bias. Using residential addresses at recruitment, we ran a comparison of our participants and non-participants, overall and by disease status, using census-based socio-economic indicators that could relate to diet. These included education, income, unemployment and the proportion of recent immigrants. Only minimal differences were observed, alleviating concerns against the possibility that selection bias could have had a strong influence on our findings. We can also rule out selection bias based on subjects’ vitamin C intake as the study was presented to potential participants as focusing on environmental factors, without specific mention of diet as one of its exposures of interest.

Our study also entails several methodological strengths. One is the population-based design of the study, which provides results that are likely to be more generalizable to the general population than those based on a selected sample participating in a randomized trial. The large sample size is also noteworthy, as this is, to our knowledge, the largest population-based study to ever evaluate the vitamin C-prostate cancer association. Another advantage is the use of incident, histologically confirmed primary prostate cancer cases. We had information on a wide range of potential confounders, including dietary, and could investigate whether associations varied according to tumor grade at diagnosis. Our study is one of the few to have investigated the role of vitamin C according to cancer aggressiveness or to have explored the role of prostate cancer detection issues in the associations under study.

The study was conducted in Montreal, Canada, where healthcare is universal and free. Although there was no screening program in place, PSA testing was frequently performed in the study population. Many subjects, cases and controls, reported five tests in the previous 5 years, suggesting that a PSA testing was likely often integrated as part of routine yearly medical exams. In situations where more than five tests were reported in the previous five years, we did not consider those in excess of five as these were probably diagnosis, not screening tests, especially for cases. Information on PSA testing allowed us to adjust for the frequency of screening, which may relate to both diet and prostate cancer diagnosis. Moreover, we were able to conduct an analysis restricting controls to subjects screened in the previous two years (76% of the control series), thereby reducing the potential for misclassification of latent cancers, a recurrent issue in both prospective and retrospective studies of this cancer type. Few studies to date assessing the role of vitamin C in prostate cancer took screening into account (Kristal et al., 1999; Cohen et al., 2000; Kirsh et al., 2006). While consideration of screening practices did not alter our findings, the ability to explore their influence on associations was an advantage over many previous investigations.

## Conclusion

This study found no association between recent dietary intakes of vitamin C and risk of overall prostate cancer, or according to disease aggressiveness. Based on this, and other available evidence, vitamin C does not seem to hold promises with regard to prostate cancer prevention.

## Author Contributions

M-EP led the conception and data acquisition of the PROtEuS study, and prepared the manuscript. HR conducted the data analysis. M-EP, HR, M-CR, and KT contributed to the interpretation of the data and critical revision of the manuscript. All authors read and approved the final version to be published.

## Conflict of Interest Statement

The authors declare that the research was conducted in the absence of any commercial or financial relationships that could be construed as a potential conflict of interest.
